# Oil and gas development exposure and atrial fibrillation exacerbation: a retrospective study of atrial fibrillation exacerbation using Colorado's all payer claims dataset

**DOI:** 10.3389/fepid.2024.1379271

**Published:** 2024-06-19

**Authors:** Lisa M. McKenzie, William B. Allshouse, Barbara Abrahams, Christine Tompkins

**Affiliations:** ^1^ Department of Environmental and Occupational Health, Colorado School of Public Health, University of Colorado Anschutz Campus, Aurora, CO, United States; ^2^ Department of Cardiology, University of Colorado School of Medicine, University of Colorado Anschutz Campus, Aurora, CO, United States; ^3^ Division of Electrophysiology, Emory University, Atlanta, GA, United States

**Keywords:** atrial fibrillation, oil and natural gas development, cohort study, environmental epidemiology, hydraulic fracturing, air pollution, noise pollution

## Abstract

**Introduction:**

Emerging risk factors for atrial fibrillation (AF) incidence and episodes (exacerbation), the most common and clinically significant cardiac arrhythmia, include air and noise pollution, both of which are emitted during oil and natural gas (O&G) well site development.

**Methods:**

We evaluated AF exacerbation risk and proximity to O&G well site development by employing a novel data source and interrupted time-series design. We retrospectively followed 1,197 AF patients living within 1-mile of an O&G well site (at-risk of exposure) and 9,764 patients living >2 miles from any O&G well site (unexposed) for AF claims in Colorado's All Payer Claims Dataset before, during, and after O&G well site development. We calculated AF exacerbation risk with multi-failure survival analysis.

**Results:**

The analysis of the total study population does not provide strong evidence of an association between AF exacerbation and proximity to O&G wells sites during (HR = 1.07, 95% CI: 0.94, 1.22) or after (HR = 1.01, 95% CI: 0.88, 1.16) development. However, AF exacerbation risk differed by patient age and sex. In patients >80 years living within 0.39 miles (2,059 feet) of O&G well site development, AF exacerbation risk increased by 83% (HR = 1.83, 95% CI: 1.25, 2.66) and emergency room visits for an AF event doubled (HR = 2.55, 95% CI: 1.50, 4.36) during development, with risk increasing with proximity. In female patients living within 0.39 miles of O&G well site development, AF exacerbation risk increased by 56% percent (95% CI: 1.13, 2.15) during development. AF exacerbation risk did not persist past the well development period. We did not observe increased AF exacerbation risk in younger or male patients.

**Discussion:**

The prospect that proximity to O&G well site development, a significant noise and air pollution source, may increase AF exacerbation risk in older and female AF patients requires attention. These findings support appropriate patient education to help mitigate risk and development of mitigation strategies and regulations to protect the health of populations in O&G development regions.

## Introduction

Atrial fibrillation (AF), the most common and clinically significant cardiac arrhythmia, impairs quality of life and substantially elevates stroke, systemic thromboembolism and heart failure risk (1, 2). The incidence and prevalence of AF are increasing (1–5). Adults aged >40 years of age have a 25% lifetime risk of developing AF (1). There are 9.3 million American's living with this chronic, dangerous, and costly condition contributing to an estimated 130,000 deaths and $6 billion in health care costs per year (6).

While knowledge on AF etiology is sparse, there are several known AF risk factors, including biological sex, advancing age, and co-morbidities (1), as well as emerging environmental risk factors including air and noise pollution (7–11). Several epidemiological studies have indicated that the risk of AF incidence increases with increasing levels of air pollutants, including particulate matter ≤2.5 micrometers, (PM_2.5_), nitrogen oxides (NO_x_), and ozone (8, 12–18), as well as higher exposure to traffic and railway noise (9, 19, 20). Additionally, studies have observed the risk of AF episodes (exascerbation) increases with increasing levels of air pollutants and noise (13, 14, 21, 22). In general, adverse cardiovascular effects are observed when audible noise levels exceed 50 A-weighted decibels (dBA) (23). Studies also suggest that nocturnal noise, which disrupts the normal sleep cycle, may be associated with greater health consequences than daytime noise (24–26). Clinically, chronic sleep deprivation is associated hypertension (27) and cardiovascular disease (28) which are firmly established and modifiable risk factors for AF (1).

One significant source of both air and noise pollution is the development of oil and natural gas (O&G) well sites. Between 2011 and 2014, 25,000–35,000 O&G well sites were developed annually in the United States (US) exceeding 150,000 total new well sites as of 2019 (29). This resulted in an extensive dispersion of O&G well sites across populated areas, with over 17 million people living within one mile of an O&G well (30). In Colorado, more than 378,000 people live within 1-mile of an O&G well site, with the densest development northeast of Denver (31). Air and noise pollution emitted during development of O&G well sites potentially impact all individuals residing near the sites (32).

As described elsewhere, modern O&G well site development is a complex, industrial process (33). Diesel-powered equipment, trucks, and generators continuously emit air pollutants and noise; on-site storage tanks, valves and pipes also emit air pollutants (34–36). Audible noise levels of 69 dBA and low frequency noise of 80 C-weighted decibels (dBC) have been reported during O&G well site development (35, 37). During development of 22-well O&G site in Colorado, 1–16 diesel trucks per hour travelled to and from the site, concentrations of PM_2.5_ more than doubled, and noise measurements exceeded 50 dBA day and night, within1,288 feet of the site (36).

It is not known if noise and air pollution emitted from O&G well site development exacerbates AF in the large and growing population living near these sites. We are not aware of any studies on this topic. However, studies indicate that living near O&G well sites may impact cardiac conditions associated with AF. Proximity to O&G well sites may affect *in-utero* heart development (38–42), increase hospitalizations for heart failure (43) in acute myocardial infarction patients (44), and increase augmentation index and blood pressure (45). Our objective is to determine if the burden of AF increases in AF patients living near O&G well site development and identify susceptible subpopulations by employing a novel time-series design and data source in a large population of AF patients using specific O&G metrics. Because air pollution and noise emissions persist in the production period following well site development, we also determine AF exacerbation increases (or persists) after the well site is developed.

## Methods

**Figure 1:**
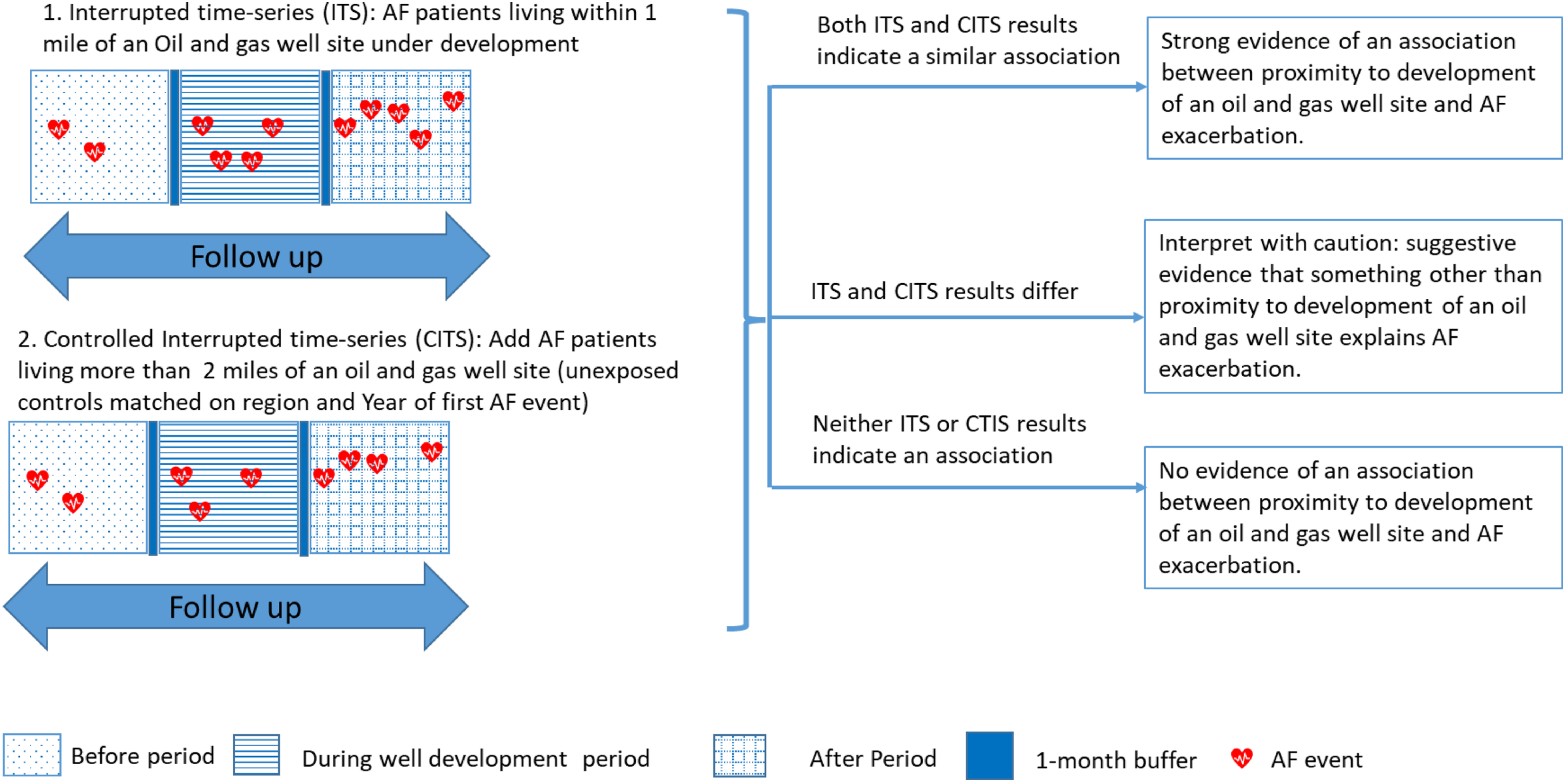
Interrupted time-series study design and assignment of before, during, and after oil and gas well site development period. AF, atrial fibrillation [adapted from Bernal et al. (47)].

We retrospectively followed 10,961 AF patients in Colorado's All Payer Claims Dataset (COAPCD) before, during, and after development of O&G well sites. Using both an interrupted time series (ITS) and controlled interrupted time series (CTIS) design (Figure 1) (46, 47), we evaluated if living near O&G well site during development exacerbates AF and if AF exacerbation persists after development of the site. We selected an ITS design because of the limited co-variate information available in Colorado's All Payer Claims Dataset (APCD). Because ITS is based on observation of a single population over time, it accounts for between group differences, such as unmeasured confounding, as well as within group characteristics that change slowly over time, secular changes, random fluctuations from one point to the next and regression to mean (46). To control for time-varying trends which do not form part of the underlying trend (e.g., seasonal, regional scale environmental events, and natural progression of AF), we also performed a CITS by adding an unexposed group as recommended by Bernal et al. (47) Per these recommendations, we included and reported results from both the ITS and CITS to provide a greater degree of confidence that an observed association between proximity to development of an O&G well site and AF exacerbation is causal (47). For example, if the CITS analysis indicates an association, but the ITS does not, then there may have been an event affecting AF in the control population that did not affect the population living within one mile. The Colorado Multiple Institutional Review Board approved our study (IRB Protocol Number 17–0692).

### Study population

We selected our cohort from the COAPCD, administered by The Center for Improving Value in Health Care. The COAPCD represents approximately 65% of Colorado's fully insured population including claims data from commercial health plans (large group, small group, and individual), Medicare, and Colorado's Medicaid Program beginning in 2009. We included patients in the COAPCD aged 18–100 years with a complete street address that we could geocode, living in a Colorado county with at least one O&G well site developed between 2010 and 2017, and at least one principal diagnosis code for AF or atrial flutter (AFl) between January 1, 2009 and December 31, 2017. From this population, we *a priori* selected patients at- risk of exposure to air and noise pollution emitted during development of an O&G site (herein referred to as exposed patients) and an unexposed population as follows.

We calculated the distance between each patient's geocoded address and the nearest O&G well site developed between 2010 and 2017 using ArcGIS Desktop 10 as described in the exposure section. We defined patients at-risk of exposure (here to in referred to as at-risk patients) as living within one mile of an O&G site based on documented noise and odor complaints, recent risk assessments, and monitoring studies indicating the potential for air and noise pollution associated with O&G well sites to impact people living within one mile (36, 48–50), as well as a robust literature supporting the use of proximity to O&G well sites as a proxy for exposure (51). Because weather, major air pollution events, and other temporal events that could exacerbate AF vary by region and AF severity may worsen over time, analysis of an location control population was necessary (47). The location control population (here to in referred to as unexposed patients) should be a population not a risk for exposure to air and noise pollution emitted from an O&G site. We selected our unexposed population from AF patients that had no O&G well sites within two miles of their home by frequency matching each at-risk patient to 13 unexposed patients by geographical region to control for regional temporal events (Supplementary Material Table S1) and year of first AF claim in the COAPCD to control for progression of AF severity. Because the spatial extent of stressors from O&G site development is not well understood and may extend beyond 1-mile, we excluded patients living 1–2 miles from an O&G site to clearly distinguish the possibility of exposure to O&G well site development stressors in at-risk patients from unexposed patients.

### Exposure

We geocoded street addresses in ArcGIS Desktop 10 using Census TIGER Address Range files from 2019 to create an address locator. For patients that could not be geocoded with ArcGIS Desktop 10, we completed a second geocoding pass with the Google Geocoding API. We obtained geocoded O&G well site locations for all O&G wells developed between 2010 and 2017, the number of wells at each well site, and the dates those wells were developed (spud date, first production date) from the Colorado Oil and Gas Information System (52).

Assuming the street address in the COAPCD is also the residential address, we temporally aligned each matched control set (up to 13 patients) to the development of the O&G well site within one mile of their matched at-risk patient's street address. We defined before, during, and after development periods as follows (Figure 1). The during development period begins on the drilling date (the spud date) of the first well on the site and ends on the first production date of the last well on the site. We then added a one-month buffer to the beginning and end of the during development period to account for well site construction activities prior to drilling and higher potential activities at the beginning of production. The before and after development periods each are equal to the length of the during development period. The before period ends at the beginning of the one-month buffer period preceding the development period. The after period begins at the end of the one-month buffer following the development period.

### Exclusions

**Figure 2:**
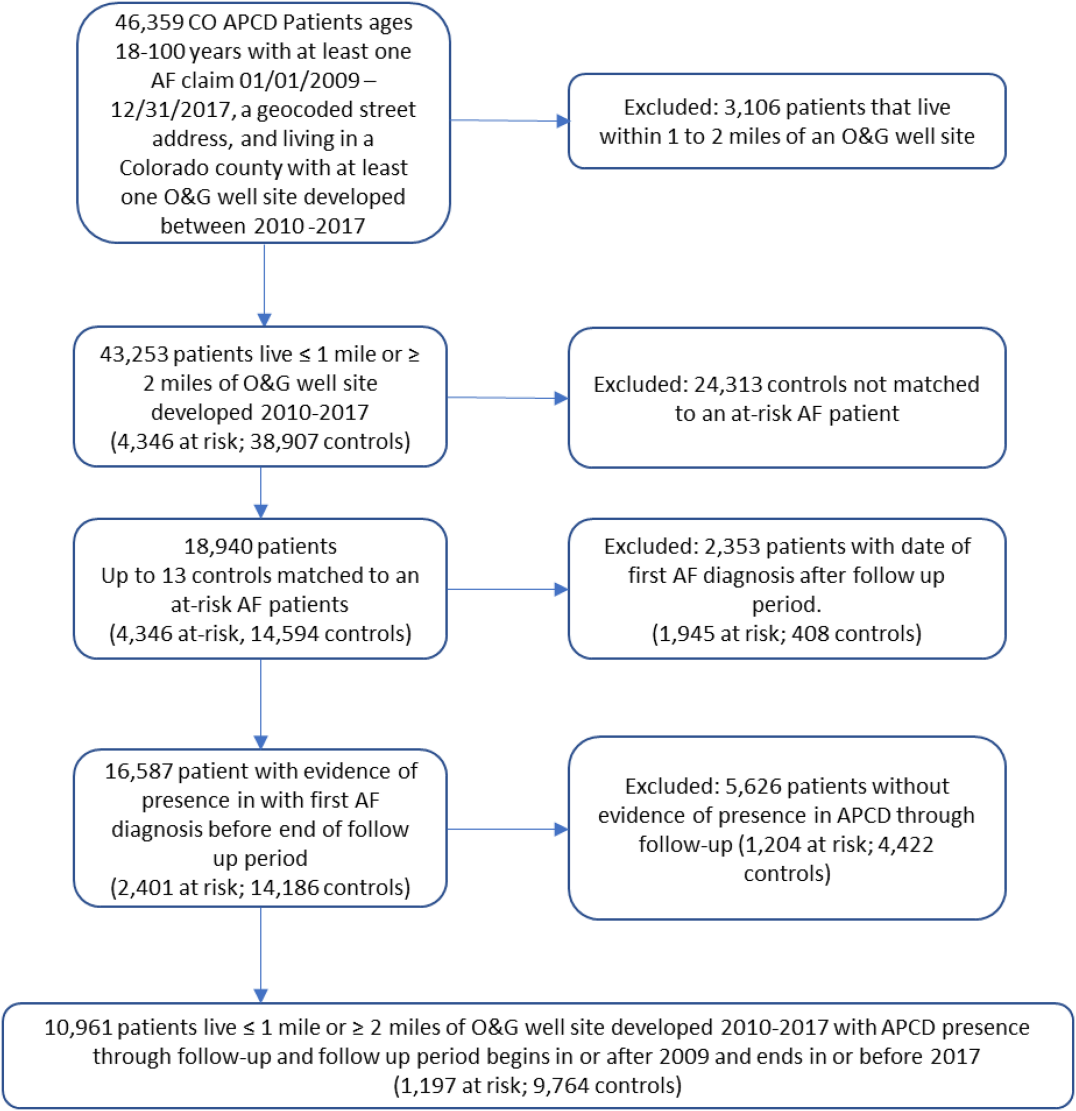
Selection of atrial fibrillation patients from Colorado all payers claim database.

We excluded patients living 1–2 miles from an O&G site (Figure 2). We next excluded 2,353 patients if the date of their first AF claim occurred later than the end of the after period because there was no evidence that the patient had AF during the follow up period. To reduce errors from unknown losses to follow up, we also excluded 5,626 patients without a claim (of any type) preceding the before period and succeeding the after period as defined in the exposure section. Our final population of 10,961 AF patients includes 1,197 at-risk and 9,764 control patients. A blinded review of claims for 1% of randomly selected at-risk and control patients confirmed 91% (90% at-risk and 94% control) patients were correctly identified as having a primary diagnosis of AF. Insufficient information was available in the remaining 9% of these patients to confirm a primary AF diagnosis.

#### Outcomes

We followed each patient from the beginning of their specific before period through the end of the after period (here to within referred as follow up) for incidence of an AF episode. We defined an AF episode as any claim, inpatient, outpatient, and emergency room, with a principal diagnostic code for AF or AFI (ICD-9-CM 427.3, or 427.31–2; ICD-10-CM I48.0–4, I48.9, I48.91, or I48.92), excluding AF diagnostic codes associated with an internal normalized ratio procedure (CPT4 85610, 93792, or R79.1). We considered occurrence of multiple AF diagnostic codes in one day or over consecutive days as one event. We also separately evaluated AF episodes associated with an emergency room visit (Supplementary Material Table S2). AF episodes that occurred in the buffer months were not counted.

#### Statistical analyses

We tested the hypothesis that there is a larger increase in incidence of AF claims during or after development of an O&G site, compared to before development, for at-risk AF patients as follows. We analyzed AF exacerbation risk with a multi-failure survival analysis by applying a Cox proportional hazard model with a robust variance estimator and clustering at the individual patient level (53, 54), using an Efron method for ties (55). We retrospectively followed each patient through their specific follow up period. We first analyzed AF exacerbation risk for only the at-risk patients (ITS) (46, 47). We then analyzed AF exacerbation risk with both the at-risk and unexposed patients (CTIS) (47), by adding an interaction term between exposure (at-risk to control referent) and period (during and after, to before referent) to our model. Parallel trend analysis indicates no difference between the exposed and unexposed populations in the before period, indicating support for the parallel assumption in CITS analysis (Supplementary Material Table S3) (56). Strong evidence of an association between proximity to development of an O&G well site and AF exacerbation is indicated if the ITS and CITS analysis yield hazard ratios (HR) of similar size (Figure 1) (47). We adjusted our model for co-variates associated with AF (biological sex, age at first AF claim in COAPCD, elevation of residence, hypertension, and diabetes) (1), and exposure (duration of well development and geographical region). We considered the direction and magnitude of individual HRs and overarching trends, based on American Statistical Association guidance (57), in both analyses.

We then stratified our population by sex, age quartiles, presence of a co-morbidity (diabetes, hypertension) and geographical region to assess whether the results between groups (e.g., male vs. female) were systematically different. Additionally, we stratified our at-risk patients into distance quartiles to assess the effect of distance from the O&G site on AF exacerbation.

We performed several sensitivity analyses. We evaluated the effect of short and long periods of well development by excluding patients with well duration periods outside the 25th—75th percentile range (75–184 days). To evaluate the impact of potential change of residence over time, we performed an analysis on patients for whom we could confirm that the street address did not change through the follow up period. Because high altitude can exacerbate AF, we performed an analysis on patients living ≤6,000 feet above sea level.

Given the small sample sizes and exploratory nature of the stratified and sensitivity analyses, no adjustments were made for multiple comparisons. All analyses were carried out using SAS 9.4 (SAS Institute, Cary, NC).

## Results

Our study population included 1,197 at-risk patients and 9,764 unexposed patients l (Table 1). At-risk patients were more likely to be male, diabetic, and hypertensive than unexposed patients. At-risk patients also had a longer duration in the COAPCD. However, the highest number of total AF claims was observed in the unexposed patients.

**Table 1:** Study population characteristics for All payer claims database patients aged 18–100 years living within one mile of an oil and gas well developed in Colorado between 2010 and 2017 or two or more miles from any Colorado oil and natural gas well site.

	At-risk: nearest well within one mile	Control: nearest well two or more miles
Total *N*	1,197	9,764
Age at first AF event in COAPCD (%)		
Greater than 80 years	27.6	28.2
74–80 years	23.5	23.8
66–73 years	30.1	27.2
<66 years	18.8	20.9
Sex (%)		
Male	53.0	50.7
Female	46.7	48.3
Missing	<1	<1
Diabetic (%)	34.9	30.5
Hypertensive (%)	83.1	77.8
Confirmed address over follow up (%)	73.2	72.2
Region		
East	89.9	89.5
Southwest	4.4	4.8
Northwest	5.7	5.6
Elevation of residence <6,500 feet	97.3	94.2
Emergency room visits (%)	30.2	30.1
Duration in COAPCD (days)		
Mean	3,023	2,983
Maximum	3,651	3,651
Minimum	605	317
Number of AF events		
Mean	14.2	13.8
Maximum	161	373
Minimum	0	0
Miles from O&G well site (*n*)		
0–0.39	299	–
>0.39–0.59	302	–
0.59–0.80	303	–
>0.8–1	293	–
Duration of O&G well site development (days)		
Mean	165	–
Maximum	844	–
Minimum	3	–

AF, atrial fibrillation; COAPCD, Colorado all payer claims dataset; O&G, oil and gas.

Table 2 presents the multi-failure survival analysis results for AF exacerbation. The analysis of the study population as a whole does not provide strong evidence of an association between AF exacerbation and proximity to O&G well site development. The ITS analysis indicates that AF exacerbation increases during (HR = 1.13, 95% CI: 0.99, 1.30) and after (HR = 1.19, 95% CI = 1.02, 1.39) well site development, compared to before well development in our total population of at-risk AF patients. With inclusion of unexposed patients in the CITS analyses this association attenuates towards the null during (HR = 1.07, 95% CI: 0.94, 1.22) and after (HR = 1.01, 95% CI: 0.88, 1.16) site development.

**Table 2:** Results from multi-failure survival analysis for AF events: hazard ratios for an AF event during and after well development periods compared to before well development.

Analysis	Interrupted time series analysis^a^		Controlled interrupted time series	
	HR during^b^ (95% CI)	HR after^b^ (95% CI)	HR during^c^ (95% CI)	HR after^c^ (95% CI)
Total population (Main analysis)	1.13 (0.99, 1.30)	1.19 (1.02, 1.39)	1.07 (0.94, 1.22)	1.01 (0.88, 1.16)
>80 years	1.41 (1.09, 1.83)	1.19 (0.93, 1.52)	1.43 (1.13, 1.81)	0.99 (0.79, 1.23
74–80 years	1.09 (0.84, 1.42)	1.16 (0.84, 1.61)	1.11 (0.86, 1.44)	1.06 (0.79, 1.40)
66–73 years	1.08 (0.84, 1.40)	1.43 (1.02, 2.0)	0.88 (0.69, 1.12)	1.10 (0.82, 1.47
<66 years	0.83 (0.61, 1.17)	0.87 (0.65, 1.17)	0.84 (0.61, 1.17)	0.87 (0.65, 1.17)
Females	1.17 (0.97, 1.42)	1.14 (0.91, 1.44)	1.21 (1.00, 1.47)	1.02 (0.82, 1.27)
Males	1.09 (0.90, 1.31)	1.25 (1.02, 1.55)	0.94 (0.79, 1.11)	1.01 (0.86, 1.20)

AF, atrial fibrillation; CI, confidence interval; HR, hazard ratio.

^a^ Does not include control patients.

^b^ Adjusted for sex, age at first AF event, elevation of residence, duration of well development, hypertension, diabetes, and region, and exposure status**.**

^c^ Adjusted for sex, age at first AF event, elevation of residence, duration of well development, hypertension, diabetes, and region, interaction between period (before, during, after development) and exposure status.

Stratified analyses indicates that age and possibly biological sex modify the risk of an AF event during O&G well site development (Table 2). In AF patients aged >80 years, ITS and CITS results for the during well development period are similar and indicate that risk for an AF event increases during well development, but not after development. In the during O&G development period, risk of an AF event increased by 43% (HR = 1.43, 95% CI: 1.13, 1.81) in at-risk patients aged >80 years. In younger patients, results attenuated towards the null. In female AF patients, ITS and CITS results for the during well development period are similar and indicate that risk for an AF event increases during well development, but not after development. The risk for an AF event in the during O&G development period increased by 21% (HR = 1.21, 95% CI: 1.00, 1.47) in at-risk female patients. No association was observed for male patients. Stratified analysis indicated that co-morbidities and region of residence did not modify risk (Supplementary Material Table S4).

**Figure 3:**
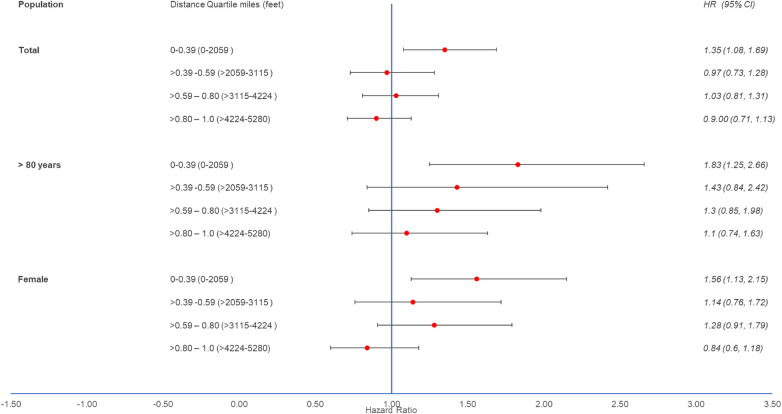
Hazard ratios with 95% confidence intervals from multi-failure survival analysis with control for total population, patients aged >80 years, and patients identified as female by distance quartile from oil and gas well development site: HRs of at-risk to controls for an atrial fibrillation event during O&G well site development compared to before development. HR, hazard ratio; CI, confidence interval.

In stratified analysis by distance quartile, ITS and CTIS results for the total population are similar and indicate increased risk of AF exacerbation during well site development in at-risk patients living within 0.39 miles (2,059 feet) and the increased risk does not persist after development of the well site. We observed a 35% increase in risk for AF events in at-risk patients living within 0.39 miles (2,059 feet) in the during well development period (HR = 1.35, 95% CI: 1.08, 1.69) (Figure 3, Supplementary Material Table S5). We did not observe associations at distances >0.39 miles for the total population. As in the main analysis, both age and sex modified the results. In patients aged >80 years living within 0.39 miles of an O&G development site, the risk of AF event increased by 83% during well development (HR = 1.83, 95% CI: 1.25, 2.66). Additionally, the results for patients aged >80 years indicate a trend of increasing risk of an AF event during well development as distance from the well site decreases and suggest the possibility of increased AF exacerbation risk up to 4,224 feet from the site. In female patients living within 0.39 miles of an oil and gas well development site, risk of AF event increased by 56% (95% CI: 1.13, 2.15) and 36% (95% CI: 0.89, 2.03) during and possibly after well development, respectively.

Table 3 presents the multi-failure survival analysis results for AF exacerbation with an emergency room visit. The analysis of the study population as a whole does not provide strong evidence of an association between AF exacerbation with an emergency room visit and proximity to O&G well site development. The ITS analysis indicates that AF exacerbation with an emergency room visit increases during (HR = 1.57, 95% CI: 0.99, 2.47) and after (HR = 1.80, 95% CI = 1.13, 2.87) well development, compared to before well development. With inclusion of unexposed control patients in the CITS analyses this association attenuates towards the null (HR = 1.11, 95% CI: 0.79, 1.56) or after (HR = 1.24, 95% CI: 0.90, 1.70) O&G well site development.

**Table 3:** Results from multi-failure survival analysis for AF event with an emergency room visit: hazard ratios for an AF event during and after well development periods compared to before well development.

Analysis	Interrupted time series analysis^a^		Parallel analysis (exposed vs. unexposed in before period)^b^	Controlled interrupted time series	
	HR during^b^ (95% CI)	HR after^a^ (95% CI)	HR (95% CI)	HR during^c^ (95% CI)	HR after^c^ (95% CI)
Total population (main analysis)	1.57 (0.99, 2.47)	1.80 (1.13, 2.87)	0.81 (0.56, 1.19)	1.11 (0.79, 1.56)	1.24 (0.90, 1.70)
>80 years	2.67 (1.26, 5.64)	1.1 (0.60, 2.01)	0.95 (0.47, 1.92)	2.55 (1.50, 4.36)	1.10 (0.60, 2.01)
74–80 years	1.20 (0.37, 3.93)	2.40 (0.82, 6.99)	0.56 (0.23, 1.40)	0.58 (0.25, 1.32)	1.34 (0.71, 2.53)
66–73 years	1.43 (0.54, 3.75)	2.71 (1.09, 6.78)	0.58 (0.27, 1.26)	0.76 (0.39, 1.47)	1.48 (0.82, 2.67)
<66 years	0.78 (0.31, 1.96)	1.11 (0.43, 2.85)	1.36 (0.66, 2.78)	0.77 (0.36, 1.64)	1.04 (0.52, 2.09)
Females	1.93 (1.0, 3.74)	1.64 (0.80, 3.37)	0.68 (0.40, 1.17)	1.30 (0.83, 2.03)	0.97 (0.59, 1.61)
Males	1.25 (0.67, 2.35)	1.94 (1.05, 3.59)	0.98 (0.58, 1.68)	0.97 (0.58, 1.61)	1.57 (1.04, 2.36)

AF, atrial fibrillation; CI, confidence interval; HR, hazard ratio.

^a^ Does not include control patients.

^b^ Adjusted for sex, age at first AF event, elevation of residence, duration of well development, hypertension, diabetes, and region, and exposure status.

^c^ Adjusted for sex, age at first AF event, elevation of residence, duration of well development, hypertension, diabetes, and region, interaction between period (before, during, after development) and exposure status.

Stratified analyses indicates that age modifies the risk of an AF event with an emergency room visits during O&G well development (Table 3). In at risk patients aged >80 years, ITS and CITS results for the during well development period are similar and indicate that risk of an AF event increases during well development. In the during well development period, risk of an AF event with an emergency room visit doubled (HR = 2.55, 95% CI: 1.50, 4.36) in at-risk patients aged >80 years. The results indicate that the risk does not persist past the well development period and show no increased risk in younger patients. Stratified analysis did not indicate biological sex, co-morbidities, or geographical region as effect modifiers (results not shown).

Sensitivity analyses excluding patients: for whom we could not confirm that the street address did not change over our follow-up period ( Supplementary Material Table S6 ), with well development durations within the 25th to 75th percentile range ( Supplementary Material Table S7 ); and living at an elevation less than 6,000 feet ( Supplementary Material Table S8 ) did not inferentially change our results.

## Discussion

Our results provide strong evidence (47) that older AF patients living within 0.39 miles (2,059 feet) of an O&G well site may experience increased AF exacerbation during site development with the possibility of increased AF exacerbation risk up to at least 0.8 miles (4,224) feet from the site, which does not persist past the well development period. Our results also suggest that AF patients identified as female living within 0.39 miles (2,059 feet) of an O&G site may experience increased AF exacerbation during site development, which does not appear to persist past the well development period. We did not observe increases in AF exacerbation in younger AF or male patients. Previous studies indicating that people living near O&G well sites may experience alterations in vascular function associated with AF (45), heart failure exacerbation (43), and increased hospitalization for acute MI (44), as well as exposures to noise and air pollution levels known to affect cardiovascular health (36) support these results. These important and biologically plausible findings contribute further epidemiological evidence that environmental stressors exacerbate AF.

Air and noise pollution emitted during the development of O&G well sites potentially impact all individuals residing in the vicinity of the sites (32). Exposure to noise elicits an acute stress reaction characterized by autonomic nervous system response, specifically, increased sympathetic activity (58), which plays an important role in the initiation and maintenance of AF (59). On the molecular level, beta adrenergic stimulation triggers an intracellular signaling cascade that can lead to intracellular calcium overload creating a particularly arrhythmogenic environment that promotes triggered activity. Resulting depolarizations generate spontaneous ectopy. Simultaneously, enhanced automaticity, promoted by increased circulating catecholamines, also leads to focal ectopic atrial activity. Both triggered activity and enhanced automaticity are believed to be the primary drivers for AF initiation. This stress reaction has been observed in response to road traffic noise (60); thus, it is plausible that exposure to stressful noise levels may induce AF in susceptible individuals. Additionally, alterations in autonomic tone, inflammation, oxidative stress, and changes in intracardiac filling pressures are known triggers for AF (58, 61–68) and are reported in response to PM_2.5_ exposure (15, 18, 69–75). Exposure to PM_2.5_ has been associated with increased blood pressure and acute alteration in vascular function, which may contribute to hypertension, an AF risk factor (1, 73, 76–79).

Interestingly, our results indicate that living near development of an O&G well site has a greater impact on older and female AF patients. Other studies also have observed that older adults living in close proximity to O&G well sites may bear greater health and mortality risks than younger adults (43, 80). Additionally, prior studies report that both women and the elderly are at higher risk of mortality and CV mortality when exposed to elevated PM_2.5_ levels (81). Our findings may be explained by age- and gender-related changes in response to physiologic stressors. Significantly higher levels of cortisol have been observed in women compared to age-matched men and older vs. younger subjects when exposed to psychological or cognitive challenges (82). It is plausible that older subjects spend more time at home, increasing the duration of exposure (83).

Our observation that AF exacerbation risk does not to persist past the well development period indicates that the increased risk is transitory in nature. A transitory increase in AF exacerbation risk could worsen AF patient acute outcomes, as evidenced by the increased risk for AF claims associated with an emergency room visit.

Our study benefited from an efficient design that accounts for unmeasured confounding, accurate definition of before, during, and after O&G well site development periods, and the availability of sequential measures of AF diagnoses and related morbidities in the COAPCD. Additionally, our temporal control design features allowed us to account for risk factors that drive AF development in an accumulating manner and time-varying variables such as season and regional air pollution events (e.g., wildfires).

Nonetheless, our study had some limitations. While our CITS design allowed us to account static environmental stressors and time varying environmental stressors a the regional level, it did not account for changing environmental stressors a the local level that may have occurred during the follow up period,, such as construction activities and development of other O&G well sites further than the closet site,. This may have biased result towards or away from the null. Assuming the street address in the COAPCD is the residential address and the possibility for change of residence in our study cohort may introduce exposure misclassification. However, our sensitivity analysis on patients for whom we could confirm the street address over the follow up period indicates that exposure misclassification from change in residential address had little effect on our results (Supplementary Material Table S6). It also is possible that some AF and co-morbidity claims were misdiagnosed. Our claim review confirmed that most (91%) patients had plausible AF diagnoses, with similar results for at-risk and control patients. Thus, this is mostly a concern for null results. Not all AF incidents may result in an COAPCD claim and not all AF patients are included in the COAPCD. Therefore, our results, may underrepresent the true incidence of AF. This too is mostly a concern for null results. It is important to appreciate that our outcome is an AF claim in the COAPCD and not new onset AF. Therefore, our results apply to the prevalence of AF. Because we did not include AF patients with addresses that could not be geocoded, our results may not be generalizable to the whole Colorado AF patient population. Because noise and air pollution measures were not available for this retrospective study, we could not elucidate specific associations between noise or air pollution and AF. Because the COAPCD includes only 65% of Colorado's population, our results may not represent the 35% of Coloradans that are uninsured or privately insured.

The prospect that proximity to O&G well site development, a significant noise and air pollution source, increases AF exacerbation risk requires attention. Health care providers should be aware of the increased risk for AF during O&G well site development for their older and female patients and provide appropriate patient education to help mitigate risk. Additionally, these findings support development of mitigation strategies and regulations to protect the health of populations living near O&G well sites. While this study advances understanding on relationships between residential proximity to development of O&G well sites and AF exacerbation, a future prospective cohort study that can follow populations for AF over the course of O&G well site development will be necessary to understand the etiological relationships between specific environmental stressors, such as noise and air pollution, and incidence and severity of AF events.

## Data Availability

The data analyzed in this study is subject to the following licenses/restrictions: The dataset was obtained from the Colorado All Payer Claims Dataset thru a data use agreement with the Center for Improving Value in Health Care. A data use agreement with Center for Improving Value in Health Care is required to access the data. Requests to access these datasets should be directed to Eddy Costa: ECosta@CIVHC.org.
