# Supplementary material for: Oil and gas development exposure and atrial fibrillation exacerbation: a retrospective study of atrial fibrillation exacerbation using Colorado's all payer claims dataset
Source: Front Epidemiol. 2024 Jun 19;4:1379271. doi: 10.3389/fepid.2024.1379271 (PMC11220195; doi:10.3389/fepid.2024.1379271)
Supplement: Supplementary file 1 [file Datasheet1.pdf]

Oil and gas development exposure and atrial fibrillation exacerbation: A retrospective study of atrial fibrillation patients in Colorado's All Payer Claims Dataset

Lisa M. McKenzie, PhD[1] William B Allshouse, PhD[1] Barbara Abrahams, MD[3] Christine Tompkins, MD[4]

SUPPLEMENTAL INFORMATION

Supplemental Table 1. Definitions for regions, diabetic, hypertension, and sleep disorders

Supplement Table 2. Counts of atrial fibrillation, ablation, electro cardioversion, and emergency room events in before, during and after development periods.

Supplemental Table 3. Results from parallel trend test: P-values for interaction between time to an AF event and exposure (at-risk and unexposed patients) in before well development period.

Supplemental Table 4. Results from multi-failure survival analysis for AF events stratified by co-morbidities and region. Hazard ratios for an AF event during and after well development periods compared to before well development.

Supplement Table 5. Results from multi-failure survival analysis stratified by distance: Hazard ratios for an AF event during and after well development periods compared to before well development.

Supplemental Table 6. Results from multi-failure survival analysis: Hazard ratios of at-risk patients to controls for an atrial fibrillation event before, during, and after well development periods with confirmed address in Colorado's All Payer Claims Database over follow up.

Supplemental Table 7. Results from multi-failure survival analysis: Hazard ratios of at-risk patients to controls for an atrial fibrillation event before, during, and after well development periods with well duration in the interquartile range (74-184 days)

Supplemental Table 8. Results from multi-failure survival analysis: Hazard ratios of at-risk patients to controls for an atrial fibrillation event before, during, and after well development periods with elevation of residence 6000 or fewer feet above sea level

Supplemental Table 1. Definitions for regions, diabetic, and hypertension

**Variable**	**Definition**
East Region	Includes Adams, Arapahoe, Boulder, Larimer, Morgan, Phillips, Sedgwick, Weld, Yuma, Cheyenne, Las Animas, and Broomfield counties
Southwest Region	Includes Archuleta, La Plata, Montezuma, and Fremont counties.
Northwest Region	Includes Garfield, Mesa, Moffat, and Jackson counties
Diabetic	ICD-9-CM 249.xx – 250.xx; ICD-10 CM E08-11, E13
Hypertension	ICD 9-CM 401.xx – 4.05.xx; ICD-10-CM I10-16

Supplement Table 2. Counts of atrial fibrillation and emergency room events in before, during and after development periods.

**Claim Type**	**Within 1 mile (exposed, n =1197))**			**More than 2 miles (unexposed n=9766)**		
	Before	During	After	Before	During	After
Atrial Fibrillation	370	406	427	2854	3165	3306
Emergency Room visit	30	41	48	250	297	291

Supplemental Table 3. Results from parallel trend test: P-values for interaction between time to an AF event and exposure (at-risk and unexposed patients) in before well development period.

Analysis	P-value of interaction between time to an AF event and exposure[1]	P-value of interaction between time to an AF event with an ER visit and exposure[1]
**Total population (Main analysis)[2]**	0.1385	0.9746
**> 80 years[2]**	0.3637	0.7619
**74 -80 years[2]**	0.6188	0.6826
**66-73 years[2]**	0.7994	0.5158
**< 66 years [2]**	0.1017	0.8906
**Females[3]**	0.1020	0.7947
**Males[3]**	0.5180	0.8132

[1]P-value ≥ 0.05 indicates parallel trends between at-risk and unexposed populations (no statistically significant difference in trends of AF events) in the before well development period.
[2]Adjusted for sex, age at first AF event, elevation of residence, duration of well development, hypertension, diabetes, and region, and exposure status.
[3]Adjusted for age at first AF event, elevation of residence, duration of well development, hypertension, diabetes, and region, and exposure status.

AF = atrial fibrillation, ER = Emergency Room

Supplemental Table 4. Results from multi-failure survival analysis for AF events stratified by co-morbidities and region. Hazard ratios for an AF event during and after well development periods compared to before well development.

	Interrupted time series analysis[1]		Controlled interrupted time series	
**Analysis**	**HR During[2] (95% CI)**	**HR After[2] (95% CI)**	**HR During[3] (95% CI)**	**HR After[3] (95% CI)**
**Hypertension**	1.13 (0.97, 1.31)	1.15 (0.97. 1.37)	1.11 (0.96, 1.28)	1.00 (0.86, 1.17)
**Diabetes**	0.94 (0.76, 1.16)	1.05 (0.83, 1.32)	1.01 (0.81, 1.25)	1.01 (0.82, 1.24)
**Eastern Region**	1.13 (0.98, 1.30)	1.21 (1.04, 1.41)	1.02 (0.89, 1.17)	0.98 (1.13)
**Southwest and Northwest Regions**	1.15 (0.78, 1.68)	1.11 (0.66, 1.86)	1.27 (0.85, 1.89)	1.12 (0.69, 1.82)

[1]Does not include unexposed patients.
[2]Adjusted for sex, age at first AF event, elevation of residence, duration of well development, hypertension, diabetes, and region, and exposure status.
[3]Adjusted for sex, age at first AF event, elevation of residence, duration of well development, hypertension, diabetes, and region, interaction between period (before, during, after development) and exposure status.

AF = atrial fibrillation, CI = confidence interval, HR = hazard ratio

Supplement Table 5. Results from multi-failure survival analysis stratified by distance: Hazard ratios for an AF event during and after well development periods compared to before well development.

Distance (miles)	Interrupted time series analysis[1]		Controlled interrupted time series	
	HR During[2] (95% CI)	HR After[2] (95% CI)	HR During[3] (95% CI)	HR After[3] (95% CI)
**Total Population**				
**0-0.39**	1.21 (0.93, 1.58)	1.16 (0.83, 1.61)	1.35 (1.08, 1.69)	1.16 (0.87, 1.53)
**>0.39 -0.59**	1.16 (0.86, 1.56)	1.25 (0.91, 1.74)	0.97 (0.73, 1.28)	0.94 (0.74, 1.19)
**0.59 – 0.80**	1.15 (0.90, 1.46)	1.24 (0.94, 1.65)	1.03 (0.81, 1.31)	1.00 (0.77, 1.30)
**>0.8 – 1**	0.98 (0.77, 1.26)	1.13 (0.86, 1.50)	0.90 (0.71, 1.13)	0.92 (0.72, 1.18)
**Patients aged > 80 years**				
**0-0.39**	1.91 (1.1, 3.29)	1.38 (0.83, 2.31)	1.83 (1.25, 2.7)	1.08 (0.78, 1.50)
**>0.39 -0.59**	1.65 (1.0, 2.74)	1.09 (0.67, 1.79)	1.43 (0.84, 2.42)	0.77 (0.51, 1.17)
**0.59 – 0.80**	1.26 (0.76, 2.1)	1.39 (0.89, 2.18)	1.30 (0.85, 1.98)	1.17 (0.76, 1.80)
**>0.8 – 1**	0.93 (0.66, 1.30)	0.96 (0.60, 1.54)	1.10 (0.74, 1.63)	0.93 (0.60, 1.45)
**Female patients**				
**0-0.39**	1.25 (0.88, 1.78)	1.25 (0.79, 1.99)	1.56 (1.13, 2.15)	1.34 (0.89, 2.03)
**>0.39 -0.59**	1.09 (0.69, 1.73)	0.79 (0.52, 1.21)	1.14 (0.76, 1.72)	0.72 (0.52, 0.99)
**0.59 – 0.80**	1.25 (0.88, 1.80)	1.19 (0.79, 1.80)	1.28 (0.91, 1.79)	1.05 (0.73, 1.51)
**>0.8 – 1**	1.04 (0.73, 1.48)	1.29 (0.86, 1.92)	0.84 (0.60, 1.18)	0.89 (0.61, 1.30)

[1]Does not include unexposed patients.

[2]Adjusted for sex, age at first AF event, elevation of residence, duration of well development, hypertension, diabetes, and region, and exposure status.

[3]Adjusted for sex, age at first AF event, elevation of residence, duration of well development, hypertension, diabetes, and region, interaction between period (before, during, after development) and exposure status.

AF = atrial fibrillation, CI = confidence interval, HR = hazard ratio

Supplemental Table 6. Results from multi-failure survival analysis: Hazard ratios of at-risk patients to controls for an atrial fibrillation event before, during, and after well development periods with confirmed address in Colorado's All Payer Claims Database over follow up.

	**Interrupted time series analysis[1]**		**Controlled interrupted time series**	
**Distance of at-risk patients from oil and gas well site (miles)**	**HR During[2] (95% CI)**	**HR After[2] (95% CI)**	**HR During[3] (95% CI)**	**HR After[3] (95% CI)**
**0-1**	1.11 (0.95, 1.29)	1.15 (0.95, 1.39)	1.13 (0.96, 1.32)	1.05 (0.88, 1.26)
**0-0.39**	1.20 (0.89, 1.63)	1.25 (0.82, 1.91)	1.35 (1.03, 1.77)	1.26 (0.87, 1.82)
**>0.39 -0.59**	1.30 (0.93, 1.88)	1.33 (0.94, 1.88)	1.12 (0.81, 1.55)	1.03 (0.78, 1.36)
**>0.59 – 0.80**	0.95 (0.71. 1.27)	1.1 (0.79, 1.53)	1.05 (0.78, 1.42)	1.10 (0.80, 1.51)
**>0.8 – 1**	1.00 (0.75, 1.33)	0.93 (0.66, 1.30)	0.97 (0.73, 1.28)	0.81 (0.60, 1.10)

[1]Does not include unexposed patients.

[2]Adjusted for sex, age at first AF event, elevation of residence, duration of well development, hypertension, diabetes, and region, and exposure status.

[3]Adjusted for sex, age at first AF event, elevation of residence, duration of well development, hypertension, diabetes, and region, interaction between period (before, during, after development) and exposure status.

AF = atrial fibrillation, CI = confidence interval, HR = hazard ratio

Supplemental Table 7 Results from multi-failure survival analysis: Hazard ratios of at-risk patients to controls for an atrial fibrillation event during, and after well development periods with well duration in the interquartile range (74-184 days)

	**Interrupted time series analysis[1]**		**Controlled interrupted time series**	
**Distance of at-risk patients from oil and gas well site (miles)**	HR During[4] (95% CI)	HR After[4] (95% CI)	HR During[5] (95% CI)	HR After[5] (95% CI)
**0-1**	1.14 (0.95, 1.36)	1.15 (0.94, 1.39)	1.06 (0.89 1.26)	1.03 (0.87, 1.22)
**0-0.39**	1.54 (1.0, 237)	1.42 (0.94, 2.15)	1.31 (0.93, 1.85)	1.18 (0.84, 1.64)
**>0.39 -0.59**	1.11 (0.77, 1.58)	1.27 (0.83, 1.94)	1.05 (0.73, 1.51)	1.17 (0.84, 1.63)
**>0.59 – 0.80**	1.16 (0.84, 1.60)	1.01 (0.70, 1.45)	1.07 (0.81, 1.42)	0.90 (0.66, 1.24)
**>0.8 – 1**	0.94 (0.69, 1.29)	1.02 (0.72, 1.44)	0.91 (0.66, 1.25)	0.95 (0.70, 1.28)

[1]Does not include unexposed patients.

[2]Adjusted for sex, age at first AF event, elevation of residence, duration of well development, hypertension, diabetes, and region, and exposure status.

[3]Adjusted for sex, age at first AF event, elevation of residence, duration of well development, hypertension, diabetes, and region, interaction between period (before, during, after development) and exposure status.

AF = atrial fibrillation, CI = confidence interval, HR = hazard ratio

Supplemental Table 8. Results from multi-failure survival analysis: Hazard ratios of at-risk patients to controls for an atrial fibrillation event before, during, and after well development periods with elevation of residence 6000 or fewer feet above sea level:

	**Interrupted time series analysis[1]**		**Controlled interrupted time series**	
**Distance of at-risk patients from oil and gas well site (miles)**	**HR During[4] (95% CI)**	**HR After[4] (95% CI)**	**HR During[5] (95% CI)**	**HR After[5] (95% CI)**
**0-1**	1.12 (0.98, 1.29)	1.18 (1.01, 1.38)	1.07 (0.93, 1.22)	1.00 (0.87, 1.16)
**0-0.39**	1.16 (0.89, 1.52)	1.14 (0.82, 1.59)	1.33 (1.05, 1.67)	1.16 (0.88, 1.54)
**>0.39 -0.59**	1.17 (0.86, 1.58)	1.26 (0.91, 1.76)	0.97 (0.73, 1.29)	0.94 (0.74, 1.19)
**>0.59 – 0.80**	1.17 (0.91, 1.51)	1.22 (0.91, 1.64)	1.04 (0.82, 1.33)	0.97 (0.74, 1.27)
**>0.8 – 1**	0.99 (077, 1.27)	1.12 (0.84, 1.49)	0.89 (0.70, 1.13)	0.90 (0.70, 1.16)

[1]Does not include unexposed patients.
[2]Adjusted for sex, age at first AF event, elevation of residence, duration of well development, hypertension, diabetes, and region, and exposure status.
[3]Adjusted for sex, age at first AF event, elevation of residence, duration of well development, hypertension, diabetes, and region, interaction between period (before, during, after development) and exposure status.

AF = atrial fibrillation, CI = confidence interval, HR = hazard ratio
